# PNA Length Restriction of Antibacterial Activity of Peptide-PNA Conjugates in *Escherichia coli* Through Effects of the Inner Membrane

**DOI:** 10.3389/fmicb.2019.01032

**Published:** 2019-05-24

**Authors:** Lise Goltermann, Niloofar Yavari, Meiqin Zhang, Anubrata Ghosal, Peter E. Nielsen

**Affiliations:** Department of Cellular and Molecular Medicine, Center for Peptide-Based Antibiotics, The Panum Institute, Faculty of Health and Medical Sciences, University of Copenhagen, Copenhagen, Denmark

**Keywords:** antisense antimicrobials, peptide nucleic acid, peptide conjugates, bacterial envelope, *Escherichia coli*

## Abstract

Peptide Nucleic Acid (PNA)-peptide conjugates targeting essential bacterial genes are showing promise as antisense antimicrobials in drug discovery. Optimization has focused on selection of target genes and exact localization around the ribosome binding site, but surprisingly a length optimum around 10–12 nucleobases has been found. Addressing this observation, we have investigated the relationship between PNA-length, PNA–RNA duplex stability and antimicrobial activity in *E. coli* in more detail. For PNAs of identical length of ten nucleobases the expected reverse correlation between the thermal stability (Tm) of the PNA–RNA duplex and the MIC for single mismatched PNAs was found. Also the expected direct correlation between the length of the PNA and the PNA–RNA duplex stability was found. Nonetheless, 10-mer PNAs [in a 6–18 mer extension series of (KFF)_3_K- and (RXR)_4_ conjugates] were the most active as antisense antimicrobials in both wild type *E. coli* MG1655 and AS19, suggesting that the size constraint is related to the bacterial uptake of PNA-peptide conjugates. This conclusion was supported by flow cytometry data showing higher bacterial uptake of shorter PNA fluorophore labeled conjugates. Interestingly, the size-limited uptake seems independent on outer membrane integrity (AS19), and thus the results suggest that the inner membrane limits the molecular size for peptide-PNA passage.

## Introduction

The extensive use of antibiotics in the past half century has given rise to development of antibiotic resistant bacterial strains by a variety of mechanisms. Recently, the threat to global human health from multi-drug-resistant bacterial infections has emphasized the urgent need for discovery of new classes of antibiotics with novel molecular targets and mechanism of action. Effective antibiotics are characterized by their specificity for bacteria and thus low toxicity toward mammalian cells. Antibiotics working via an antisense mechanism, targeting essential bacterial genes is one way of ensuring high specificity. Peptide nucleic acids (PNAs) are particularly well-suited as such antimicrobial antisense agents because of their resistance to nucleases and proteases as well as favorable sequence specific RNA hybridization properties, although bacterial uptake is a general challenge in the application of PNAs (and of oligonucleotides and their analogs and mimics in general) as antisense agents. However, conjugation to bacteria penetrating peptides (BPPs) was discovered as a successful way to ensure increased PNA uptake and thereby efficacy (Good et al., 2001). Specifically, the (KFF)_3_K peptide conjugated to a PNA targeting the essential bacterial gene, *acpP*, was shown to be sequence specifically bactericidal against *Escherichia coli* (Good et al., 2001). Subsequently, antibacterial effects of peptide-PNA [and also of phosphordiamidate morpholino oligomer (PMO) (Geller et al., 2018)] conjugates against a range of antibiotic resistant bacterial species have been reported, e.g., multi-resistant *Klebsiella pneumoniae* (Kurupati et al., 2007) and *Haemophilus influenzae* (Otsuka et al., 2017), and *Pseudomonas aeruginosa* (Montagner et al., 2017) in planktonic cultures as well as biofilms. Furthermore, recent studies have identified the inner-membrane SbmA protein as a necessary transporter of some peptide-PNA conjugates (Ghosal et al., 2013). However, other carrier peptides do not require SbmA for activity, and SbmA is therefore not the only mechanism of transport across the inner membrane (Ghosal et al., 2013; Hansen et al., 2016).

Antisense efficacy relies on high sequence specific affinity for the mRNA target, and the strongest antisense effect is achieved by targeting sequences around and proximally upstream of the translation start codon (Dryselius et al., 2003). In general, increased efficacy with increased RNA target affinity, and thus length to a first approximation, is expected. However, early studies have surprisingly revealed an optimum PNA (and PMO) size of 10–12 nucleobases for antimicrobial antisense agents (Good et al., 2001; Deere et al., 2005).

It is worth noting that – analogously to the situation in eukaryotic cells – antisense agents may exert their activity via different mechanisms, broadly operating by mRNA degradation via RNaseH activation or by steric blockage of mRNA translation or processing. Antisense targeting in bacteria have almost exclusively been performed using PNA and PMO oligomers which do not activate RNaseH and therefore appear to work primarily through translation inhibition, although some reports do also indicate that mRNA decay (possibly induced by translation blockage) may result. RNA silencing occurs naturally in bacteria and these pathways may eventually also be exploited for RNA targeted antibiotic discovery (Soler Bistué et al., 2009; Good and Stach, 2011; Lopez et al., 2015).

In this study we have more systematically investigated the influence of PNA-length on the antimicrobial effect in *E. coli* using a series of peptide-PNA antibacterial antisense agents targeting *acpP* through translation inhibition with the aim of elucidating the mechanism behind the puzzling PNA length limitation. This was done for different carrier peptides, in different *E. coli* strains and in the presence or absence of the SbmA inner membrane transporter.

## Materials and Methods

### Strains and PNAs

*Escherichia coli* strains MG1655, MG1655(Δ*sbmA*) (Ghosal et al., 2013), AS19 (Sekiguchi and Iida, 1967), ATCC25922(Δ*rfaG*) (Ebbensgaard et al., 2018), and *Klebsiella pneumoniae* ATCC 13883 were used throughout the study and cultured in non-cation adjusted Muller-Hinton Broth (MHB) (Sigma-Aldrich, cat.no. 70192) at 37°C.

Peptide nucleic acids (Table 1) were dissolved in water and the concentration determined using a NanoDrop spectrophotometer at 260 nm. Low binding plastics (Axygen, Corning) were used throughout.

**Table 1:** Peptide-PNA-conjugates used (mm = mismatch).

PNA	Peptide	PNA sequence	Length	Target
2301	–	CTC ATA CTC T	10	*acpP*
4223	–	TG CTC ATA CTC T	12	*acpP*
4224	–	A GTG CTC ATA CTC T	14	*acpP*
4226	–	CG ATA GTG CTC ATA CTC T	18	*acpP*
3961	–	CTC **T**TA C**A**C T	10	mm for PNA2301
4521	–	TG CTC **T**TA C**A**C T	12	mm for PNA4223
4522	–	A GTG CTC **T**TA C**A**C T	14	mm for PNA4224
4524	–	CG ATA GTG CTC **T**TA C**A**C T	18	mm for PNA4226
4720	H-(KFF)_3_K-eg1-	C**A**CATACTCT	10	mm for PNA2108
4721	H-(KFF)_3_K-eg1-	CTC**T**TACTCT	10	mm for PNA2108
4722	H-(KFF)_3_K-eg1-	CTCATA**G**TCT	10	mm for PNA2108
4723	H-(KFF)_3_K-eg1-	CTCATACT**G**T	10	mm for PNA2108
5077	H-(KFF)_3_K-eg1-	TA CTC T	6	*acpP*
5079	H-(KFF)_3_K-eg1-	C ATA CTC T	8	*acpP*
5080	H-(KFF)_3_K-eg1-	TC ATA CTC T	9	*acpP*
2108	H-(KFF)_3_K-eg1-	CTC ATA CTC T	10	*acpP*
5082	H-(KFF)_3_K-eg1-	G CTC ATA CTC T	11	*acpP*
5083	H-(KFF)_3_K-eg1-	TG CTC ATA CTC T	12	*acpP*
5164	H-(KFF)_3_K-eg1-	A GTG CTC ATA CTC T	14	*acpP*
5166	H-(KFF)_3_K-eg1-	ATA GTG CTC ATA CTC T	16	*acpP*
5168	H-(KFF)_3_K-eg1-	CG ATA GTG CTC ATA CTC T	18	*acpP*
5396	H-(KFF)_3_K-eg1-	TAC **A**CT	6	mm for PNA5077
5270	H-(KFF)_3_K-eg1-	C**T** TAC **A**CT	8	mm for PNA5079
5271	H-(KFF)_3_K-eg1-	TC**T** TAC **A**CT	9	mm for PNA5080
5272	H-(KFF)_3_K-eg1-	C TC**T** TAC **A**CT	10	mm for PNA2108
5273	H-(KFF)_3_K-eg1-	GC TC**T** TAC **A**CT	11	mm for PNA5082
5274	H-(KFF)_3_K-eg1-	TGC TC**T** TAC **A**CT	12	mm for PNA5083
5404	H-(KFF)_3_K-eg1-	AG TGC TC**T** TAC **A**CT	14	mm for PNA5164
5406	H-(KFF)_3_K-eg1-	A TAG TGC TC**T** TAC **A**CT	16	mm for PNA5166
5408	H-(KFF)_3_K-eg1-	CGA TAG TGC TC**T** TAC **A**CT	18	mm for PNA5168
3986	H-(R-Ahx)_6_-(β-Ala)-	CTC ATA CTC T	10	*acpP*
4227	H-(R-Ahx)_6_-(β-Ala)-	TG CTC ATA CTC T	12	*acpP*
4228	H-(R-Ahx)_6_-(β-Ala)-	A GTG CTC ATA CTC T	14	*acpP*
4229	H-(R-Ahx)_6_-(β-Ala)-	ATA GTG CTC ATA CTC T	16	*acpP*
4230	H-(R-Ahx)_6_-(β-Ala)-	CG ATA GTG CTC ATA CTC T	18	*acpP*
3987	H-(R-Ahx)_6_-(β-Ala)-	C TC**T** TAC **A**CT	10	mm for PNA3986
4500	H-(R-Ahx)_6_-(β-Ala)-	TGC TC**T** TAC **A**CT	12	mm for PNA4227
4501	H-(R-Ahx)_6_-(β-Ala)-	AG TGC TC**T** TAC **A**CT	14	mm for PNA4228
4502	H-(R-Ahx)_6_-(β-Ala)-	A TAG TGC TC**T** TAC **A**CT	16	mm for PNA4229
4503	H-(R-Ahx)_6_-(β-Ala)-	CGA TAG TGC TC**T** TAC **A**CT	18	mm for PNA4230
4099	H-(R-Ahx-R)_4_-Ahx-(β-Ala)-	CTC ATA CTC T	10	*acpP*
4246	H-(R-Ahx-R)_4_-Ahx-(β-Ala)-	TG CTC ATA CTC T	12	*acpP*
4247	H-(R-Ahx-R)_4_-Ahx-(β-Ala)-	A GTG CTC ATA CTC T	14	*acpP*
4248	H-(R-Ahx-R)_4_-Ahx-(β-Ala)-	ATA GTG CTC ATA CTC T	16	*acpP*
4288	H-(R-Ahx-R)_4_-Ahx-(β-Ala)-	CG ATA GTG CTC ATA CTC T	18	*acpP*
5631	H-(KFF)_3_K-eg1-Cys(BODIPY)-	TA CTC T	6	*acpP*
5491	H-(KFF)_3_K-Cys(BODIPY)-	CTC ATA CTC T	10	*acpP*
5629	H-(KFF)_3_K-eg1-Cys(BODIPY)-	A GTG CTC ATA CTC T	14	*acpP*

*The PNAs were synthesized as previously reported (Good et al., 2001; Ghosal et al., 2013). eg1, 8-amino-3,6-dioxaoctanoic acid; Ahx, 6-aminohexanoic acid; Cys, cysteine; BODIPY, 4,4-difluoro-4-bora-3a,4a-diaza-s-indacene. Underlined: 10 nucleobase common motif. Bold and underlined: mismatched nucleobases.*

### Tm Determination

Thermal stability (Tm) measurements were performed on a Cary 300 Bio UV-visible spectrophotometer (Varian, Cary, NC, United States) connected to a temperature controller. Thermal melting profiles were obtained in 10 mM Na-phosphate (pH 7.0) containing 0.1 mM EDTA and 100 mM NaCl using a heating range of 5–95°C at a rate of 0.5^o^C/min. The melting temperature (*T*m) was determined from the maximum of the first derivative of the heating curve. Cuvettes of 1.0 cm path length and 1.0 ml volume were used for all experiments. RNA oligonucleotide 5089: 5’-AGA GUA UGA GCA CUA UCG-3’ was used for all Tm experiments.

### MIC Determination

MIC values were determined by broth microdilution according to standard protocols with a few modifications (Cockerill et al., 2012). An overnight bacterial cell culture was diluted to approximately 5 × 10E5 CFU/ml in non-cation-adjusted MHB. 190 ul bacterial solution was dispensed into a low-bind 96-well plate (Thermo-Scientific, cat.no. 260895) along with 10 ul of the test compound. The plate was incubated in a Tecan Genios plate reader at 37°C for 18 h with linear shaking, OD was measured every 20 min at 595 nm. The MIC was determined as the lowest concentration, which inhibited visible growth in the wells (OD(595 nm) < 0.1).

### Spheroplast Preparation

*Escherichia coli* cells were cultured in MHB overnight, diluted to OD(595 nm) = 0.3 and incubated at 37°C with shaking for 1–2 h. *E. coli* cells were harvested in exponential phase at 7000 g, 8 min at 4°C, washed twice in 0.01M Tris–HCl, pH 7.4 and then resuspended in the same buffer containing 0.5M sucrose. Then lysozyme was added to the cell suspension at a final concentration of 150 μg/ml and EDTA was added to a final concentration of 10 mM. This suspension was incubated overnight in a water bath at 37°C. Spheroplasts were gently washed three times in 0.01 M Tris–HCl with 0.5 M sucrose (Birdsell and Cota-Robles, 1967). Spheroplasts were incubated with peptide-PNA for 2 h, serially diluted with 0.5 M sucrose in 0.01 M Tris–HCl, pH 7.4, plated on LB agar plates, and colonies were counted after over-night incubation.

### Flow Cytometry

*Escherichia coli* cells were cultured in MHB overnight and diluted 100× into fresh media and grown to exponential phase at OD_595_ = 0.2. The cells were pelleted and re-suspended (to a 100× dilution) in PBS buffer containing the PNA, and then incubated for 1 h at room temperature. The cell suspension was diluted five times in PBS and profiled using an Apogee Flow Cytometer A10.

## Results

### MIC-PNA Length Relation of (KFF)_3_K-PNA Conjugates

**FIGURE 1:**
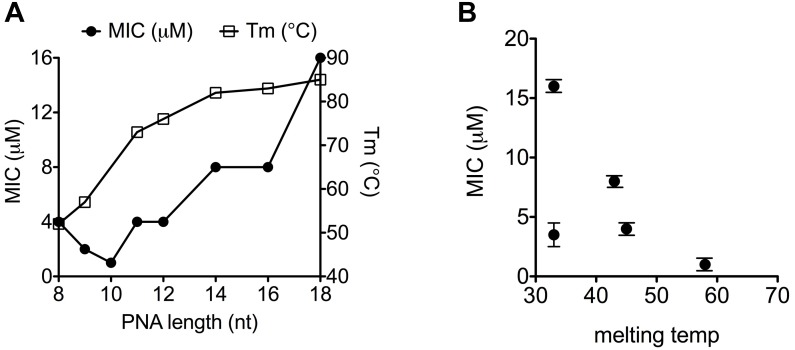
Thermal stability of PNA–RNA duplex correlates with MIC-value. **(A)** MIC and melting temperature for (KFF)_3_K-PNAs of increasing PNA length. **(B)** Correlation between melting temperature and MIC for (KFF)_3_K-PNA with single base mismatches.

Exploiting the most commonly used *E. coli* gene target as well as delivery BPP, the antimicrobial effect of a series of 8–18 mer (KFF)_3_K-PNA conjugates against the *acpP* target in *E. coli* as well as the Tm of their duplexes with complementary RNA was determined (Table 2 and Supplementary Figure S1). The results (Figure 1A) revealed a direct correlation between antimicrobial activity and Tm for PNAs shorter than 10 nucleobases, while a reverse correlation was seen for PNAs longer than 10, thus showing an activity optimum at 10 nucleobases against *E. coli* MG1655 in agreement with previous reports (Good et al., 2001; Deere et al., 2005). Importantly, when analogous mismatch PNA constructs were tested, low activity and no length dependence was found (Table 2 and Supplementary Figure S2); thus corroborating the conclusion that the antimicrobial activity is indeed caused by an antisense mechanism of action also for the PNAs of different lengths. This observation extended to *Klebsiella pneumonia* (Table 2) in which the *acpP* sequence target is conserved demonstrating that the length constraint is not limited to *E. coli*.

**Table 2:** MIC-values.

(KFF)_3_K	PNA5079 8-mer		PNA5080 9-mer		PNA2108 10-mer		PNA5082 11-mer		PNA5083 12-mer	
	Match	mm	Match	mm	Match	mm	Match	mm	Match	mm
MG1655	4	*16*	2–4	*16*	0.5–1	*16*	4	*16*	4	*16*
MG1655(Δ*sbmA*)	16	*16–32*	16	*16*	8–16	*16–32*	16	*16*	16	*16*
AS19	0.5	*2*	0.5	*2*	0.125	*1–2*	0.5	*2*	0.5	*2*
ATCC25922 (Δ*rfa*G)	0.25	>*8*	nd	nd	0.125	*4*	>0.25	>1	1–2	*1–2*
*Klebsiella pneumoniae*	8–16	>*32*	nd	nd	2	>*32*	nd	nd	8	>*32*

*MIC values (μM) of 8–12-mer match or mismatch (italicized) anti-acpP PNA conjugate. nd, Not determined.*

### Correlation Between PNA–RNA Duplex Stability and Antibacterial Activity

From the results of a previous sequence target optimization study, a general relationship between antibacterial efficacy and PNA–DNA duplex stability was not apparent (Dryselius et al., 2003). However, due to confounding effects of mRNA target position, a direct comparison of these two parameters requires the location of the mRNA target to remain constant while only changing the antisense oligomer (e.g., in terms of nucleobase sequence). By introducing single mismatches at different positions in a well-described antisense peptide-PNA conjugate, and exploiting the differential effect of mismatch position on duplex destabilization, we interrogated the correlation between PNA/RNA affinity, as measured by thermal melting, and the antimicrobial effect of the PNA. In this case a clear, direct correlation between PNA/RNA duplex stability (melting temperature) and the antisense efficacy (as measured by MIC) was observed (Figure 1B and Supplementary Table S1) (except for one outlier, which must be caused by an unexplained biological effect). Therefore the above described effect of PNA length on antibacterial activity (Figure 1A) follows the expected duplex stability between 8 and 10 nucleobases, but reverses above 10 nucleobases. Consequently, the decreasing activity as the PNA is extended beyond 10 nucleobases must be due to another phenomenon, of which length dependent decreased uptake would be an obvious candidate, as previously proposed but not experimentally supported (Good et al., 2001).

### PNA Length Effects in Envelope Mutants

The length dependence was also studied in *E. coli* AS19 and Δ*rfaG*, which because of a compromised LPS barrier are inherently more sensitive toward antisense PNAs, and antibiotics in general (Good and Nielsen, 1998). The AS19 strain has a not fully characterized truncated LPS structure (Sekiguchi and Iida, 1967), while the Δ*rfaG* strain lacks the entire outer LPS core (Ebbensgaard et al., 2018). Similar to the results obtained with the MG1655 strain, the (KFF)_3_K-PNA with a PNA-length of 10 nucleobases showed the lowest MIC value, and any decrease or increase in PNA-length reduced the growth inhibitory effect of the (KFF)_3_K-PNA (Table 2). This suggests that the inner membrane (or the peptidoglycan cell wall) is a significant contributor to the length constraints. In order to eliminate the effect of the carrier peptide, we also tested the length dependence of naked PNA in the AS19-strain. Increasing the PNA length from 10- to 12- and 14-mer reduced activity in AS19 (Table 3 and Supplementary Figure S3), indicating that even without a carrier peptide, increasing the PNA length impairs antibacterial efficacy.

**Table 3:** MIC-values.

Naked PNA	PNA2301	10-mer	PNA4223	12-mer	PNA4224	14-mer
AS19	2	>*32*	4	*16*	32	>*32*

*MIC values (μM) of 10–14-mer match or mismatch (italicized) anti-acpP PNA in AS19.*

### Effect of the SbmA Transporter

The antibacterial activity of (10-mer) (KFF)_3_K-PNA is dependent on the inner membrane transporter SbmA for activity (Ghosal et al., 2013). Therefore, a reduced uptake of longer PNAs could be due to length (size) limiting transport efficacy of SbmA. As expected, none of the (8- to 12-mers) (KFF)_3_K-PNAs exhibited any antisense related antibacterial activity in the MG1655(Δ*sbmA*) strain since match and mismatch compounds showed no significant difference in MIC values (Table 2). However, it has previously been demonstrated that the (KFF)_3_K-peptide can be substituted with other peptides [such as H-(R-Ahx)_6_-(β-Ala) or H-(R-Ahx-R)_4_-Ahx-(β-Ala)] resulting in PNA conjugates that do not require SbmA for bacterial uptake (Ghosal et al., 2013; Hansen et al., 2016). Thus, we investigated whether the length effect was limited to the (KFF)_3_K peptide by measuring the MIC values of the anti-*acpP*-PNA conjugated to the H-(R-Ahx)_6_-(β-Ala) or the H-(R-Ahx-R)_4_-Ahx-(β-Ala) peptide, respectively. Again, exceeding a PNA length of 10 nucleobases reduced the MIC value significantly (Table 4), indicating that this effect is not limited to the (KFF)_3_K-PNA conjugate. Combined, these results suggest that PNA length is important for transport through SbmA as well as for transport via an SbmA-independent pathway.

**Table 4:** MIC values (μM) of H-(R-Ahx)_6_-(β-Ala)-PNA and H-(R-Ahx-R)_4_-Ahx-(β-Ala)-PNA from 10-mer to 18-mer.

H-(R-Ahx-R)_4_-Ahx-(β-Ala)-	PNA3986 10-mer	PNA4227 12-mer	PNA4228 14-mer	PNA4229 16-mer	PNA4230 18-mer
MG1655	1–2	8	>32	>32	>32
MG1655(Δ*sbmA*)	1	4	>32	>32	>32
AS19	0.5/*2*^∗^	1/*1–2*^∗^	2/*nd*^∗^	2/*2*^∗^	2/*2–4*^∗^

**H-(R-Ahx)_6_-(β-Ala)-**	**PNA4099 10-mer**	**PNA4246 12-mer**	**PNA4247 14-mer**	**PNA4248 16-mer**	**PNA4288 18-mer**

MG1655	1	8	>16	>16	>16
MG1655(Δ*sbmA*)	1	4	>32	>32	>32

*^∗^Mismatch PNA.*

### Effect of PNA on *E. coli* Spheroplasts

**FIGURE 2:**
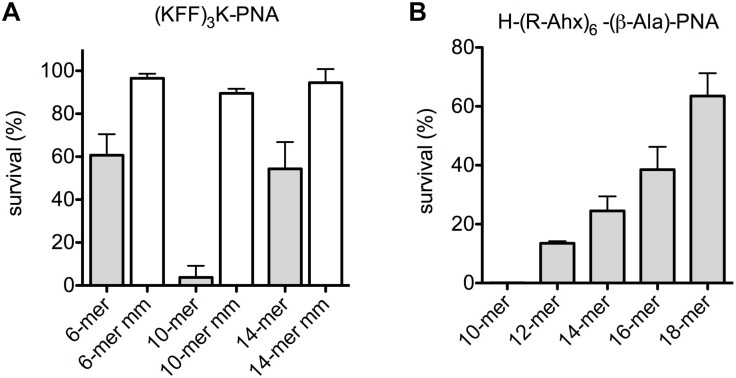
Uptake of PNA is limited by the inner membrane in *E. coli* spheroplasts. Survival of *E. coli* spheroplasts after incubation with peptide-PNA conjugates **(A,B)** of different PNA length.

In an effort to completely abolish any contribution of the outer membrane, the effect of anti-*acpP*-PNAs of different lengths on *E. coli* spheroplasts revival was measured. Spheroplasts were isolated and incubated with peptide-PNA conjugates of different lengths, and survivors were determined by plating (Figure 2). The 10-mer was clearly the most efficient in reducing bacterial survival among the 6-, 10- and 14-mer (KFF)_3_K-PNAs (Figure 2A). Analogously, the 10-mer H-(R-Ahx-R)_4_-Ahx-(β-Ala)-PNA showed higher activity than the longer PNAs (Figure 2B), and a reverse activity/length relation was seen in line with the data obtained with intact cells.

### Determination of Peptide-PNA Uptake by Flow Cytometry

**FIGURE 3:**
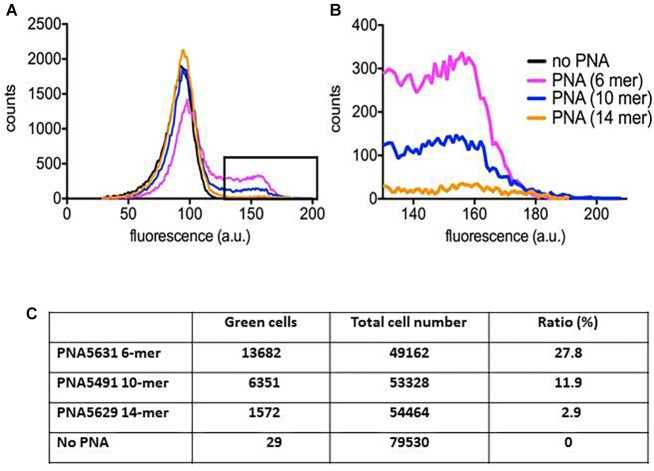
Peptide-PNA uptake monitored by flow cytometry. *E. coli* culture incubated with BODIPY fluorophore labeled peptide-PNA with different PNA length was profiled by flow cytometry. **(A)** Full profiles of *E. coli* cultures incubated with each of the three peptide-PNA conjugates or without PNA. Background fluorescence extends to approx. 130 fluorescence units. **(B)** Excerpt from (**A**, box) showing only the part of the bacterial population containing the labeled peptide-PNA-conjugate. **(C)** Percentage of bacterial cells with peptide-PNA uptake.

In order to physically monitor the uptake of the peptide-PNA-conjugates of different PNA length, we constructed BODIPY-labeled variants, which could be traced using flow cytometry. Although inclusion of fluorophores generally hampers cellular activity (increases MIC) (unpublished results), the 10-mer retained higher activity compared to the 6-mer and 14-mer, respectively (Figure 3). Although the major part of the bacterial population contained only a limited amount of PNA (Figure 3A), a fraction with a very significant uptake of the green BODIPY-labeled PNA could be detected as a tail of higher fluorescence on the histogram (Figure 3B). Similar population heterogeneity of uptake at low concentration of antimicrobial peptides has previously been reported (Pérez-Peinado et al., 2018). Analyzing the fraction of green cells reveals a reverse correlation between the PNA length and bacterial cellular uptake, although the conjugates containing the 6-mer and 14-mer PNA show similar activity (MIC). Thus by compensating for the different uptake efficiency, a very rough estimate of the intracellular efficacy of the 6-, 10- and 14-mer PNA indicates a 1:3:10 ratio between these. This supports the hypothesis that longer PNAs are less efficiently taken up thereby limiting their otherwise higher intrinsic potency, and that the optimum for a 10-mer PNA reflects a balance between cellular uptake efficiency and intrinsic antisense activity.

## Discussion

Uptake of antimicrobial agents through the Gram-negative outer membrane is determined by their size, symmetry and hydrophobicity. In general, small ( < 600 Da) hydrophilic or amphiphilic molecules, such as beta-lactams and some quinolones, penetrate the outer membrane through porins (Hancock and Bell, 1988; Nikaido, 2003), while certain larger antibiotics may be able to enter through nutrient transporters (Braun et al., 2001).

Larger and more hydrophobic compounds (e.g., aminoglycosides) are generally taken up much slower through a self-promoted pathway in which the compounds accumulate on the cell surface until reaching a critical concentration at which, pores form in the outer membrane allowing a surge of antibiotic to enter (Hancock and Bell, 1988; Richter and Hergenrother, 2019).

Other compounds, such as the bacteriocins enter by an energy dependent pathway using the tolA or tonB systems (Nikaido, 2003). Highly hydrophobic compounds are usually ineffective against Gram-negatives except for deep rough mutants in which these antibiotics can diffuse through the lipid bilayer, also known as the hydrophobic pathway (Nikaido, 1976; Hancock and Bell, 1988). Furthermore, perturbation of the LPS-layer such as that found in rough or deep-rough mutants generally increases the sensitivity toward hydrophobic antibiotics while reducing the sensitivity to selected hydrophilic antibiotics, probably because of rearrangement of the membrane and reduction of the number of porins (Sen and Nikaido, 1991).

The PNA-peptide conjugates having masses of around 5 kDa are significantly larger than conventional antibiotics and also somewhat larger than most naturally derived antimicrobial peptides. It is therefore unlikely that porins are involved in transport over the outer membrane. This is supported by the fact that no porin mediated resistance mechanisms have yet been identified for PNA-peptide conjugates.

The BPP-PNAs differ from membrane-disruptive cationic antimicrobial peptides by having a cytoplasmic target and therefore must also cross the peptidoglycan cell wall and the inner membrane. Other antibiotic classes such as the aminoglycosides, the macrolides and some quinolones are most likely sufficiently hydrophobic to pass through the inner membrane by passive diffusion, and the cell wall appears permeable for particles up to at least 2 nm (Demchick and Koch, 1996). Hydrophilic molecules such as most antimicrobial peptides with intracellular targets utilize transporters in the inner membrane for translocation (such as SbmA) (Paulsen et al., 2016). We have previously described a similar mechanism for certain peptide-PNA conjugates, which do exploit the SbmA-transporter for passage. For PNAs conjugated to arginine-rich carrier peptides, however, it remains unclear how the inner membrane is traversed. No porin or transporter mutants have yet been identified, which could provide resistance toward PNAs conjugated to arginine-rich peptides. This suggests that no single non-essential gene product is responsible for the uptake and/or that multiple pathways exist for entry into the cytoplasm for these compounds, and the mechanism may rely on local disturbance/disruption of the lipid bilayer. Finally, it is unlikely that the peptidoglycan cell wall constitutes a size barrier for the longer PNA conjugates, as particles of 2 nm, i.e., much wider than the diameter of the PNA oligomer, transverse freely through the cell wall (Demchick and Koch, 1996).

## Conclusion

The present results clearly show that antisense potency of PNA-peptide conjugates in *E. coli* exhibits an optimum around a target size of 10 nucleobases, and that this optimum is due to opposing PNA length effects on mRNA binding affinity versus efficiency of bacterial uptake. Interestingly, the size-limited uptake is independent of the delivery peptide and the data indicates that the size limitation may predominantly be ascribed to restrictions of inner membrane passage. Thus further studies elucidating the detailed molecular mechanism for bacterial uptake is warranted in order to understand the details of the uptake mechanism as well as to facilitate rational approaches for design of novel delivery vehicles that may relax this size-limitation thereby allowing the development of longer and thus higher potency bacterial antisense agents.

## Author Contributions

PN and LG designed the experiments. LG, NY, AG, and MZ performed the experiments. LG and PN wrote the manuscript.

## Conflict of Interest Statement

The authors declare that the research was conducted in the absence of any commercial or financial relationships that could be construed as a potential conflict of interest.
