# Supplementary material for: PNA Length Restriction of Antibacterial Activity of Peptide-PNA Conjugates in Escherichia coli Through Effects of the Inner Membrane
Source: Front Microbiol. 2019 May 24;10:1032. doi: 10.3389/fmicb.2019.01032 (PMC6542938; doi:10.3389/fmicb.2019.01032)
Supplement: Supplementary file 1 [file Data_Sheet_1.PDF]

Supplementary Material

Table S1. MICs and melting temperatures for *acpP*-targeting PNAs with single base mismatches.

PNA	Target RNA (*acpP*) 3'-ACGAGTATGAGAAT	MIC (µM)	Tm (°C)
2108	H-(KFF)$_3$K-eg1-TCATACTCT	**1**	52,0
4720	H-(KFF)$_3$K-eg1-**A**CATACTCT	**4**	45,0
4721	H-(KFF)$_3$K-eg1-TC**T**TACTCT	**16**	33,0
4722	H-(KFF)$_3$K-eg1-TCATA**G**TCT	**4**	33,0
4723	H-(KFF)$_3$K-eg1-TCATACT**G**T	**8**	43,2

Figure S1

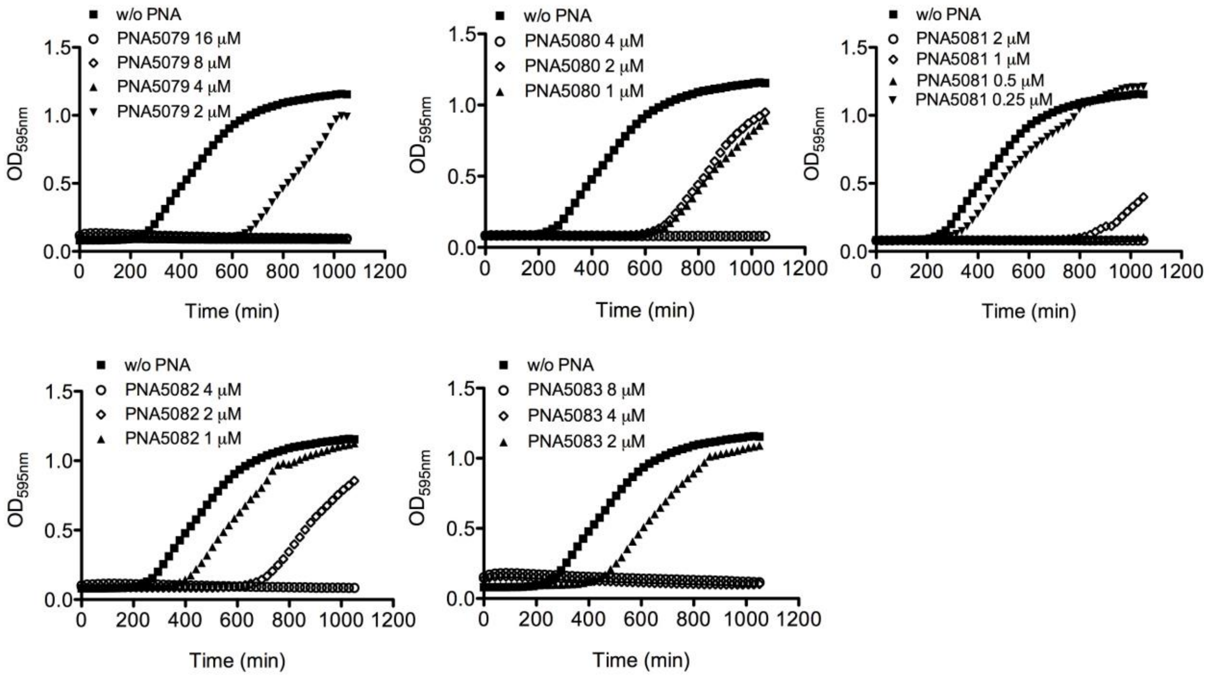

Figure S1. MIC determination. Growth curves for *E.coli* MG1655 with different lengths (8-12-mer) of (KFF)$_3$K-eg1-PNA. OD was recorded at 595 nm over 18h.

Figure S2

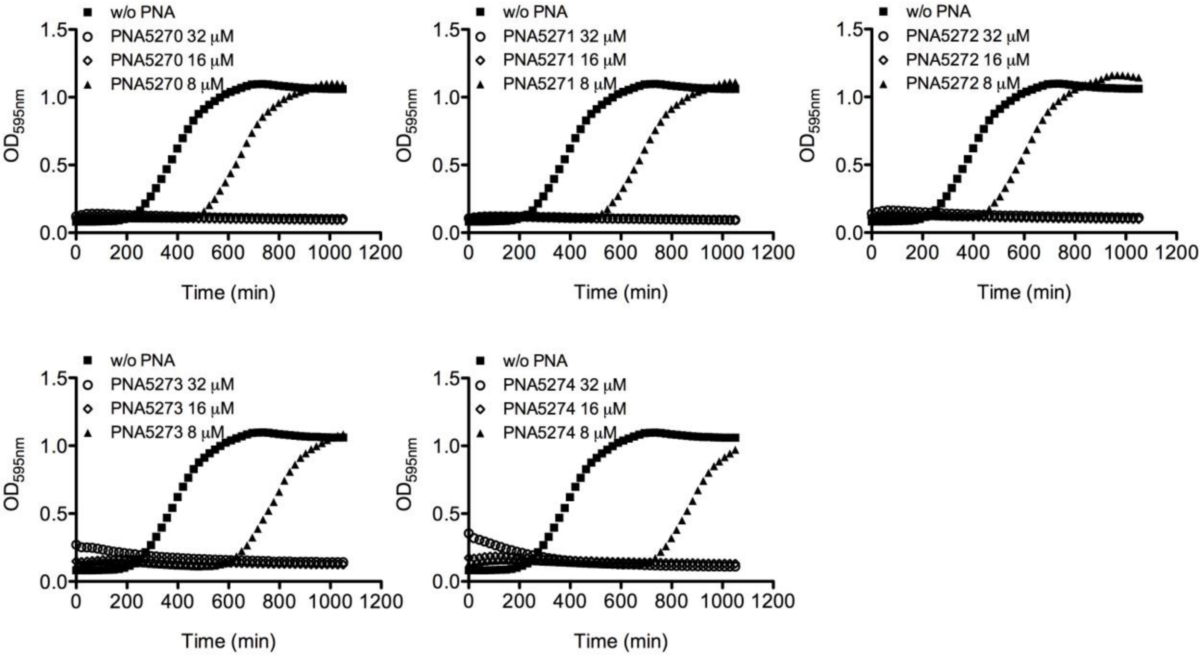

Figure S2. MIC determination. Growth curves for *E.coli* MG1655 with different lengths of mm $(KFF)_3$K-eg1-PNA. OD was recorded at 595 nm.

Figure S3

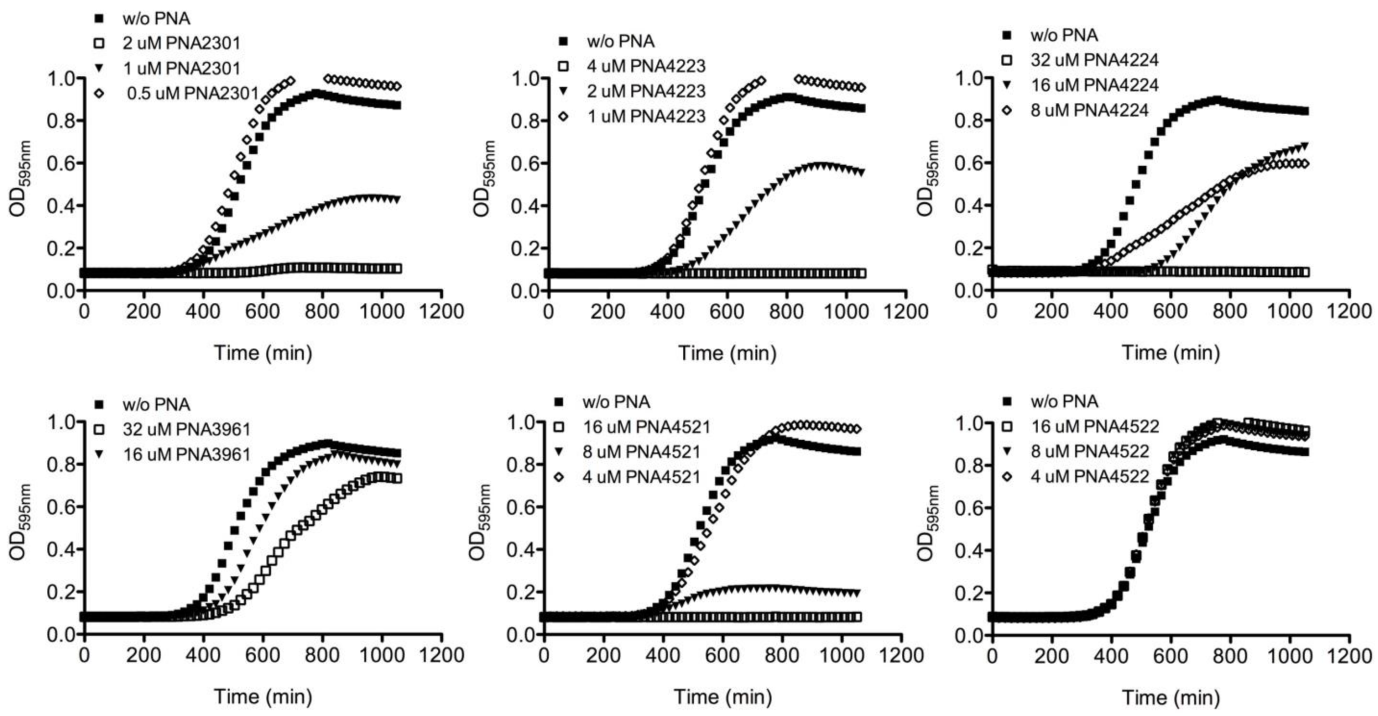

Figure S3. MIC determination of naked PNA, 10-mer, 12-mer and 14-mer match and mismatch in *E.coli* AS19. Growth was measured at 595nm over 18h.
